# COVID-19 Outbreak, Senegal, 2020

**DOI:** 10.3201/eid2611.202615

**Published:** 2020-11

**Authors:** Ndongo Dia, Ndeye Aïssatou Lakh, Moussa Moise Diagne, Khardiata Diallo Mbaye, Fabien Taieb, Ndeye Maguette Fall, Mamadou Alioune Barry, Daye Ka, Amary Fall, Viviane Marie Pierre Cisse Diallo, Oumar Faye, Mamadou Malado Jallow, Idrissa Dieng, Mamadou Ndiaye, Mamadou Diop, Abdoulaye Bousso, Cheikh loucoubar, Marie Khemesse Ngom Ndiaye, Christophe Peyreffite, Louise Fortes, Amadou Alpha Sall, Ousmane Faye, Moussa Seydi

**Affiliations:** Institut Pasteur, Dakar, Senegal (N. Dia, M.M. Diagne, F. Taieb, M.A. Barry, A. Fall, Oumar Faye, M.M. Jallow, I. Dieng, M. Diop, C. Ioucoubar, C. Peyreffite, A.A. Sall, Ousmane Faye, M. Seydi);; Service des Maladies infectieuses de l'hôpital Fann, Dakar (N.A. Lakh, K.D. Mbaye, N.M. Fall, D. Ka, V.M.P.C. Diallo, L. Fortes);; Ministère de la Santé et de l’Action Sociale (MSAS), Dakar (M. Ndiaye, A. Bousso, M.K.N. Ndiaye)

**Keywords:** COVID-19, SARS-CoV-2, severe acute respiratory syndrome coronavirus 2, viruses, respiratory infections, zoonoses, coronavirus disease, Senegal

## Abstract

The spread of severe acute respiratory syndrome coronavirus 2 began later in Africa than in Asia and Europe. Senegal confirmed its first case of coronavirus disease on March 2, 2020. By March 4, a total of 4 cases had been confirmed, all in patients who traveled from Europe.

The spread of severe acute respiratory syndrome coronavirus 2 (SARS-CoV-2) was delayed in Africa and Latin America. The earliest recorded case of coronavirus disease (COVID-19) in Africa was identified in Egypt 7 weeks after the beginning of the outbreak (*1*). On February 28, 2020, Nigeria declared the first confirmed case in sub-Saharan Africa (*2*). On March 2, Senegal confirmed an imported case, then 2 additional imported cases the next day, and a fourth on March 4.

In Senegal, the Ministry of Health coordinated all standard operating procedures (SOPs) for the detection, notification, case management, and transport of persons with suspected COVID-19 cases from entry points (e.g., airport, harbor), healthcare centers, or locality to the referral service, using the initial WHO case definition (*3*). A nasopharyngeal swab specimen was collected from any symptomatic suspected case-patient or person in contact with confirmed case-patients for SARS-CoV-2–specific real-time RT-PCR testing at the Institut Pasteur Dakar (IPD) (Appendix). Samples were accompanied by a standardized investigation form collecting demographical information, clinical details, and history of exposure (contact with a confirmed case or history of travel).

In the case of a positive diagnosis of SARS-CoV-2 infection, an active surveillance of contacts or co-exposed persons was initiated immediately around the index case. The nasopharyngeal swabs of positive patients were used for the next-generation sequencing.

Senegal experienced its first COVID-19 suspected case on February 26. During February 26–March 4, a total of 26 suspected case-patients (14 female and 12 male) were tested for a possible SARS-CoV-2 infection. Patient age range was 3–80 years (mean 35.16 years; median 33 years). Of the 26 suspected case-patients, 2 male and 2 female were confirmed as SARS-CoV-2 infected; they were 34 (patient 1), 82 (patient 2), 68 (patient 3), and 33 (patient 4) years of age. Because all were probably infected outside of the country, they were reported as imported cases. They all arrived by airplane, 3 from France and 1 from England. One case-patient had traveled manifesting symptoms undetected by the crew members. Patients 2 and 3, a married couple, traveled together; both had diabetes and hypertension, and both experienced mild clinical symptoms. All 4 patients were admitted to the Isolation and treatment Center (ITC) established by the Ministry of Health (MoH) in Dakar, Senegal. They all were apyretic the first day of hospitalization; they required mild supportive care but not oxygen therapy. In the adopted protocol, discharge of a patient from ITC required 2 consecutive negative tests for SARS-CoV-2 taken 48 hours apart. Patient 1 was discharged after 4 days, and patient 4 after 7 days, whereas patients 2 and 3 stayed for 16 days. Indeed, the viral shedding lasted longer with patients 2 and 3, the oldest. Patients 1 and 4 represented a moderate risk for dissemination of the disease, but patients 2 and 3 represented a high risk for diffusion. Investigations of contact cases and swabbing of high-risk contact cases have not to date identified any secondary cases. 

We successfully obtained the complete genome sequences from the 4 SARS-CoV-2–positive patients’ samples. The 4 complete genomes were nearly identical across the whole genome; sequence identity was >99%. Outside of the stretch of 44 undetermined nucleotides (19360–19403) in the genome of the strain from patient 1, only 1 nucleotide difference was mapped in open reading frame 8 of patient 4’s virus isolate genome, at position 28259 with a T®C synonymous substitution in virus isolate genomes from patients 1, 2, and 3. All genome sequences have been deposited in GISAID database (https://www.gisaid.org; accession nos. EPI_ISL_418206–9).

Phylogenetic analyses revealed that SARS-CoV-2 strains from Senegal clustered with strains from diverse origins (Europe, Asia, Latin America, and Africa). Of note, they were close to the hCoV-19 Netherlands Haarlem 1363688 2020 EPI ISL 413572 and hCoV-19 Taiwan NTU03 2020 EPI ISL 413592 strains. The strains from Senegal clustered together, as shown by the phylogenetic branch with a high bootstrap value of 99% (Figure). All strains from Senegal belong to the ORF8-L isoform.

**Figure:**
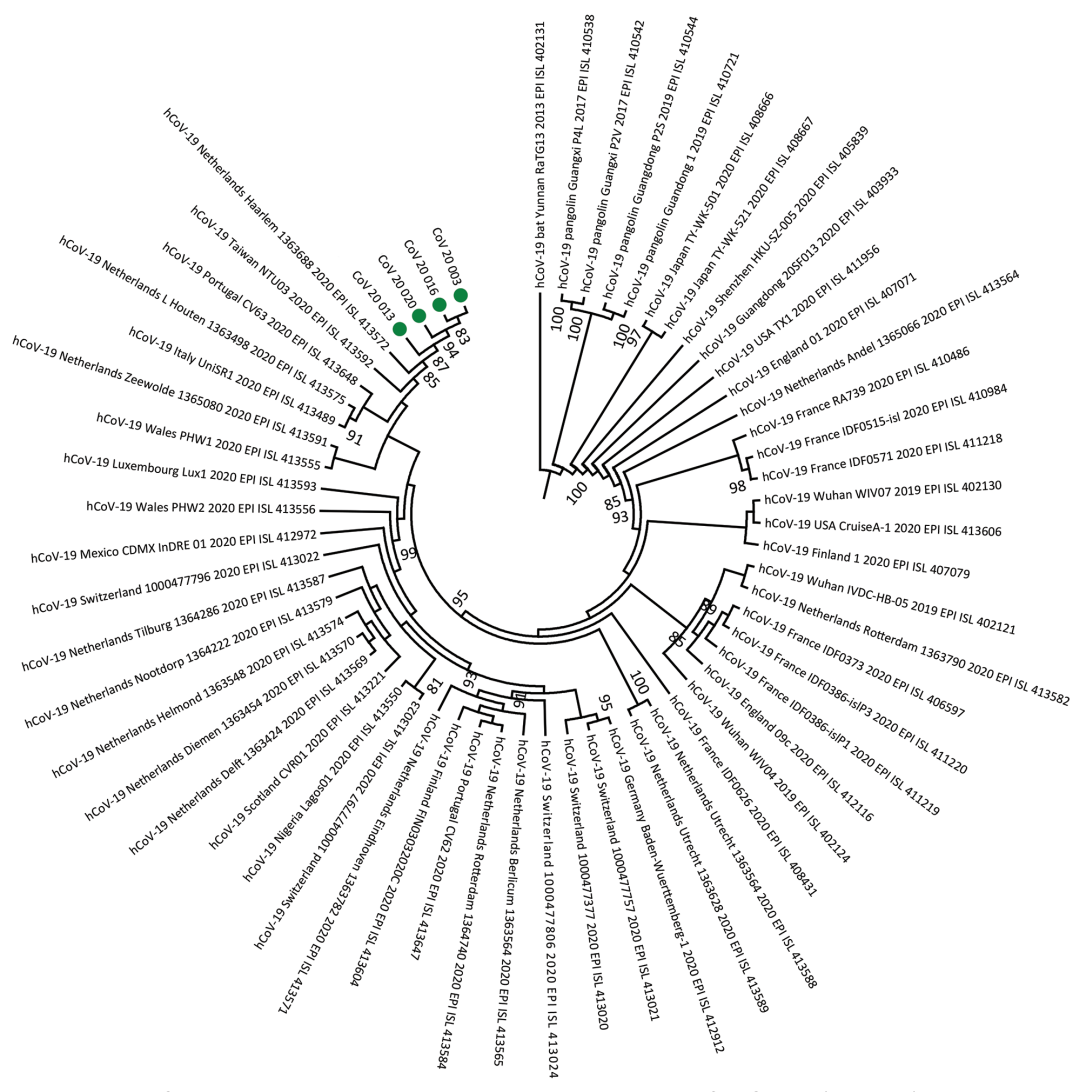
Phylogeny of 4 severe acute respiratory syndrome coronavirus 2 strains isolated from Senegal (green dots). Whole-genome nucleotide sequences were compared with 56 other genome sequences from the coronavirus disease pandemic retrieved from GenBank and GISAID (https://www.gisaid.org) databases. Sequences were aligned with MAFFT (https://mafft.cbrc.jp/alignment/server). We generated the phylogenetic tree by the maximum-likelihood method under the HKY85-gamma nucleotide substitution model using IQ-TREE (http://www.cibiv.at/software/iqtree). We assessed robustness of tree topology with 1,000 replicates; bootstrap values >75% are shown on the branches of the consensus trees. Phylogenetic analyses revealed that strains from Senegal clustered with strains from diverse origins (Europe, Asia, Latin America, and Africa). CoV, coronavirus; hCoV, human coronavirus.

The diagnosis of these cases showed the surveillance system of Senegal’s capacity to quickly detect, isolate, and investigate those cases to take adequate control measures. Our findings indicate that the earliest cases in Senegal or sub-Saharan Africa were imported from Europe, implying that the particularly high volume of direct flights from Europe was a key factor in the spread of the virus in West Africa. However, we cannot exclude the possibility that a few COVID-19 cases were missed at that time in Senegal, including paucisymptomatic or asymptomatic cases (*4**,**5*). Our study emphasizes the imperative need for efficient epidemiologic investigations to identify the cases and characterize the transmission modes to prevent, control, and stop the spread of COVID-19.

Appendix Additional information about the COVID-19 outbreak in Senegal.
