# Supplementary material for: COVID-19 Outbreak, Senegal, 2020
Source: Emerg Infect Dis. 2020 Nov;26(11):2771–3. doi: 10.3201/eid2611.202615 (PMC7588548; doi:10.3201/eid2611.202615)
Supplement: Appendix — Additional information about the COVID-19 outbreak in Senegal. [file 20-2615-Techapp-s1.pdf]

Article DOI: https://doi.org/10.3201/eid2611.202615

COVID-19 Outbreak, Senegal, 2020

Appendix

Material and Methods

COVID-19 Senegalese Surveillance System

In Senegal, the Ministry of Health (MoH) coordinated all standard operating procedures (SOP) for the detection, notification, case management, and transport of suspected coronavirus disease (COVID-19) cases following the initial case definition from the World Health Organization (*1*). MoH established an alert committee with emergency hotline. In accordance with the surveillance protocol, physicians suspecting a COVID-19 case must immediately contact the alert committee in charge to evaluate whether the patient is a possible case (for symptomatic and contact cases). Once the suspicion is confirmed, the patient is then isolated for 14 days at the Fann district university hospital, and biological samples are collected and shipped to the referral laboratory, Institut Pasteur Dakar (IPD) fulfilling the biosafety and biosecurity regulations for Senegal.

A nasopharyngeal swab specimen was collected from symptomatic suspected case or person in contact with confirmed cases and placed in universal viral transport medium (Becton Dickinson, https://www.bd.com), stored at 4-8° C, and transported to IPD within 24 hours of collection for testing. Once in IPD Respiratory Virus National Reference Center Laboratory, specimens were processed immediately for SAR-SCoV-2–specific real-time RT-PCR detection, or kept at +4°C until the next morning if delivered after 8 p.m. At least 2 aliquots of each sample were also stored at –80°C for biobanking or additional analysis. Samples were accompanied by a standardized investigation form collecting demographical information, clinical details, and history of exposure (history of travel or contact with a confirmed case) filled by the clinicians.

In the case of a positive diagnosis of severe acute respiratory syndrome coronavirus 2 (SARS-CoV-2) infection, an active surveillance of contacts or co-exposed persons was initiated immediately around the index case.

SARS-CoV-2 Diagnosis

The initial extraction steps were carried out in a BSL-3 laboratory. Total RNA was extracted from 200 µL of nasopharyngeal swab using a QIAmp Viral RNA Mini Kit (QIAGEN, https://qiagen.com) according to the manufacturer's specifications. RNAs were eluted with 60 µl nuclease-free water and immediately used for testing. An inactivation step was added: shortly after addition of the lysis buffer, the mixture was heated at 65°C during 30 min for viral inactivation.

For the SARS-CoV-2 diagnostic, 4 protocols were used: the Berlin protocol (*2*) along with the TIBMolBiol kits (https://www.tib-molbiol.com), which targeted the *E*, *N* and *RdRP* genes; the HKU protocol (*3*), targeting the *N* and *RdRP* genes; and the protocol proposed by the Unité des Virus Emergents in Marseille (distributed by European Virus Archive Global) targeting the *E* gene. All protocols were validated using the Superscript III one step RT-PCR system with Platinum Taq Polymerase (Invitrogen, https://www.thermofisher.com) in a final volume of 20 µL including 5 µL of extracted RNA. The cycling conditions for all protocols were as follows: 50°C for 15 min and 95°C for 3 min, followed by 45 cycles of 95°C for 15 s and 60°C for 1 min.

According to IPD algorithm, at this very early stage of the outbreak, a sample was tested simultaneously with the TIBMolBiol targets (*E* and *RdRP*) and the Marseille target (*E* gene). The Berlin protocol (*E*, *N,* and *RdRP*) or HKU protocols were used for additional confirmation of SARS-CoV-2 positive samples if necessary.

Genome Sequencing and Phylogeny

The primary nasopharyngeal swab samples of the first 4 positive patients were directly used for next-generation sequencing. Briefly, for each sample, 11µL of RNA extracts were reverse transcribed using the IV RT kit (Invitrogen) and the amplicons were obtained using tiled, multiplexed primers designed by ARTIC network (https://artic.network/ncov-2019). The sequencing library preparation was carried out using the DNA Flex Library kit (Illumina Technologies, https://www.illumina.com) and Nextera DNA UD indexes (Illumina). The concentrations of libraries were measured using a Qubit dsDNA High Sensitivity kit on a Qubit 3.0 fluorometer (ThermoFisher). Then, a pool of 60 pm was loaded into an Iseq 100 system (Illumina) for sequencing. Each sample was processed in duplicate to increase the success of

getting a complete genome sequence and to confirm the quality output. Dry lab analysis was then performed using IPD's local servers. After removing host (human) reads, assembly per alignment using Bowtie2 version 2.0.6 software (https://sourceforge.net/projects/bowtie-bio/files/bowtie2/2.0.6) was done in order to construct contigs. The corresponding fasta files were merged using Emboss merger (BLAST, www.ncbi.nlm.nih.gov).

Nucleotide sequences of Senegalese SARS-CoV-2 strains and genome sequences from the current COVID-19 pandemic retrieved from NCBI (http://www.ncbi.nlm.nih.gov/genbank) and GISAID (https://www.gisaid.org) databases (as of March 14, 2020) were used for phylogenetic analysis. Sequences were aligned with MAFFT. The phylogenetic tree was generated using the maximum-likelihood (ML) method under the HKY85-gamma nucleotide substitution model using IQ-TREE. Robustness of tree topology was assessed with 1,000 replicates.

References

1. World Health Organization. Global surveillance for human infection with novel coronavirus (2019-nCoV): interim guidance, 21 January 2020 [cited 2020 Sep 16]. https://www.ephi.gov.et/images/20200121-global-surveillance-for-2019-ncov.pdf

2. Corman VM, Landt O, Kaiser M, Molenkamp R, Meijer A, Chu DK, et al. Detection of 2019 novel coronavirus (2019-nCoV) by real-time RT-PCR. Euro Surveill. 2020;25. https://doi.org/10.2807/1560-7917.ES.2020.25.3.2000045

3. Chu DKW, Pan Y, Cheng SMS, Hui KPY, Krishnan P, Liu Y, et al. Molecular diagnosis of a novel coronavirus (2019-nCoV) causing an outbreak of pneumonia. Clin Chem. 2020;66:549–55. https://doi.org/10.1093/clinchem/hvaa029

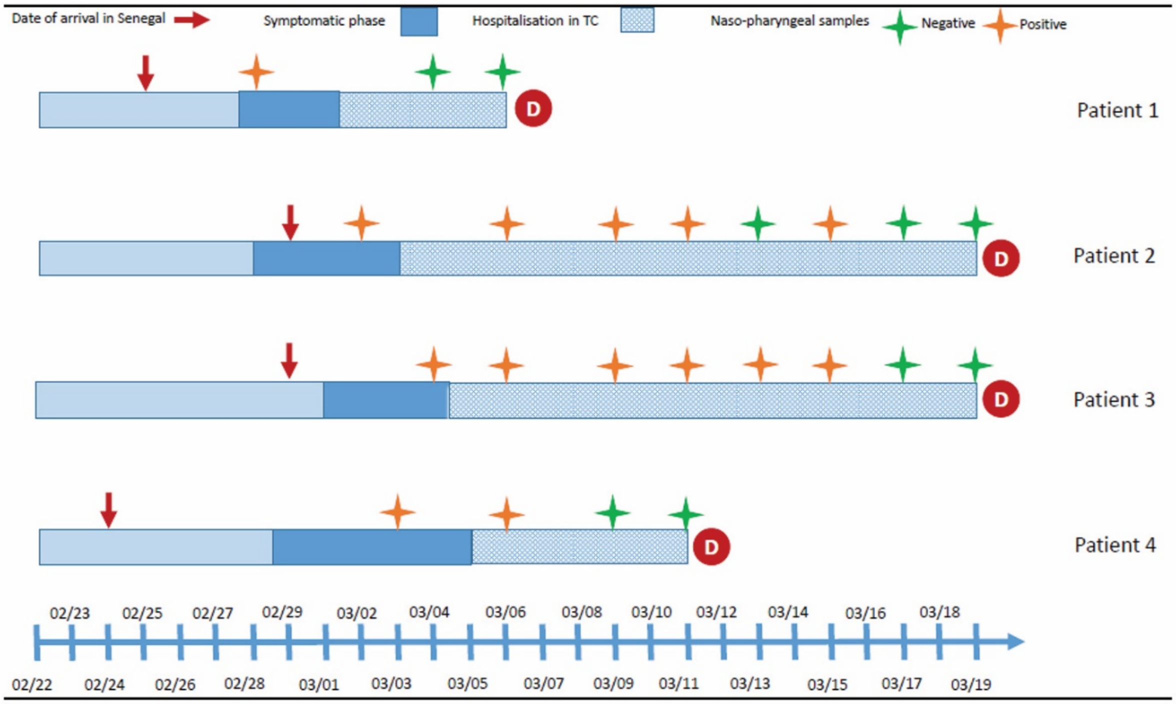

Appendix Figure. Schematic description of the course of the 4 COVID-19 cases in Senegal. For each patient, detailed information for their arrival in Senegal and clinical trajectory of the disease are shown, including symptomatology, hospitalization, timeline of results from nasopharyngeal swabs through the course of the disease, and discharge.
